# Unveiling the STAT3-ACC1 axis: a key driver of lipid metabolism and tumor progression in non-small cell lung cancer

**DOI:** 10.7150/jca.93890

**Published:** 2024-03-04

**Authors:** Hong Zhai, Tiansheng Zheng, Lihong Fan

**Affiliations:** 1 Department of Respiratory Medicine, Shanghai Tenth People's Hospital, Tongji University School of Medicine, Shanghai200072, China.; 2 Department of Integrated Traditional Chinese and Western Medicine, Shanghai Tenth People's Hospital, Tongji University School of Medicine, Shanghai, China.

**Keywords:** Lung Cancer, Lipid Metabolism, STAT3, ACC1

## Abstract

**Background and Objective:** Lung cancer is a prevalent global malignancy, and investigating the metabolic reprogramming of tumor cells has significant therapeutic implications. This study aims to explore the molecular mechanism driving the progression of non-small cell lung cancer (NSCLC), with a specific emphasis on the STAT3-ACC1-FAS axis involved in fatty acid synthesis.

**Methods:** The levels of Signal transducer and activator of transcription 3 (STAT3) and acetyl-CoA carboxylase 1 (ACC1) were determined in mouse NSCLC specimens and cell lines using Western blot and qPCR methods. Various assays such as CCK-8, colony formation, EDU, wound-healing, and transwell migration were employed to assess cancer cell proliferation, migration, and invasion. Additionally, a nude mouse xenograft model was utilized for *in vivo* tumor growth analysis. The interaction between STAT3 and ACC1 was examined through chromatin immunoprecipitation and dual-luciferase assays.

**Results:** The study observed upregulation of STAT3 and ACC1 in NSCLC tissues. Notably, the suppression of STAT3 and ACC1 inhibited the *in vitro* progression and lipid synthesis of NSCLC cells. Furthermore, STAT3 enhanced lipid synthesis by upregulating ACC1 expression. Mechanistic assays revealed that this process occurred through direct activation of ACC1 transcription by STAT3. STAT3 played a vital role in regulating lipid metabolism and supporting NSCLC progression.

**Conclusion:** The findings of this study underscore the significance of the STAT3-ACC1-FAS axis in NSCLC. The activation of ACC1 through STAT3-mediated transcription serves as a crucial mechanism for stimulating the progression of NSCLC tumors and promoting lipid synthesis. Consequently, targeting the STAT3-ACC1 axis may present a promising avenue for the diagnosis and treatment of NSCLC patients.

## Introduction

Lung cancer stands as the leading cause of cancer incidence and mortality worldwide 1. Non-small cell lung cancer (NSCLC), in particular, commands specific attention due to its high prevalence and resistance to conventional therapies 2, 3. Consequently, there is an urgent need to unravel the intricate molecular events governing NSCLC progression in order to develop more effective treatment strategies.

Recent advancements in cancer research have underscored the pivotal role of metabolic reprogramming in the development and progression of tumors, including non-small cell lung cancer (NSCLC) 4-6. Among various metabolic pathways, lipid metabolism has emerged as a critical component in supporting the rapid proliferation and migration of cancer cells, encompassing lipid acquisition for energy, de novo synthesis of fatty acids (FASyn), and fatty acid β-oxidation 7, 8.

Fatty acid synthesis (FASyn) is a well-established metabolic feature linked to tumor cells 9, with its initial step being regulated by the rate-limiting enzyme known as acetyl-CoA carboxylase (ACC) 10. Currently, there are two identified isoforms of acetyl-CoA carboxylase: ACC1, located in the cytoplasm and primarily responsible for regulating fatty acid synthesis, and ACC2, found in the mitochondria and mainly involved in controlling fatty acid oxidation 11. Various studies have demonstrated that ACC1 is the predominant isoform in different types of human lung cancer cells, with minimal detection of ACC2 12. ACC1 plays a crucial role in FASyn by catalyzing the ATP-dependent carboxylation of acetyl-CoA, resulting in the formation of malonyl-CoA, which is subsequently utilized for fatty acid production through consecutive reactions. Cerulenin, a non-competitive inhibitor of FAS, obstructs the condensation of malonyl-CoA with acetyl-CoA, thereby inhibiting fatty acid synthesis and inducing apoptosis in cancer cells 13, 14. Inhibiting ACC1 has been shown to significantly decrease cell proliferation and trigger apoptosis in prostate and breast cancer cells 15, 16. Therefore, targeting FASyn has been proposed as a potential therapeutic approach, especially considering the upregulation of ACC1 mRNA in various cancer types. Consequently, ACC1 represents an attractive therapeutic target for intervention.

Signal Transducer and Activator of Transcription (STAT) proteins have a critical role in regulating a wide range of metabolic processes. They transduce signals from metabolites, cytokines, growth factors, and receptors, thereby controlling tumor progression and treatment resistance 17. Moreover, STAT proteins govern cancer and immune cell metabolism, as well as multiple facets of mitochondrial activity, including energy metabolism and lipid-mediated maintenance of mitochondrial integrity 18. Due to their pivotal role in regulating metabolic states 19, STAT proteins have emerged as promising therapeutic targets for modifying the reprogramming of cancer metabolism. However, the exact regulatory role of STAT3 in fatty acid synthesis and the correlation between STAT3 and ACC1 in NSCLC remain uncertain.

The aim of this study was to introduce a novel aspect by investigating the interaction between STAT3 and ACC1, hypothesizing that this interplay crucially modulates lipid metabolism in NSCLC, impacting cell proliferation and migration. This exploration offered fresh insights into the metabolic mechanisms of NSCLC and potential targeted therapeutic approaches.

## Methods and Materials

### Antibodies and reagents

The subsequent antibodies were commercially obtained as follows: mouse anti-STAT3 (9139S, CST), rabbit anti-ACC1 (A19627, ABclonal), goat anti-rabbit IgG (H+L), HRP conjugated (SA00001-2, Proteintech), goat anti-mouse IgG (H+L), HRP conjugated (SA00001-1, Proteintech), and HRP-conjugated beta actin (HRP-60008, Proteintech).

### Animals

To generate lung epithelial cell conditional Kras mutation (Kras^G12D/+; p53fl/fl^), we performed a crossbreeding between Kras^G12D/+^ mice and p53^fl/fl^ mice. The LSL-Kras^G12D^ allele, generously provided by Dr. Tyler Jacks' laboratory, has been thoroughly described in previous studies. To activate the mutant Kras, we administered adenoviral Cre intratracheally to LSL-Kras mice, following an established protocol. Specifically, the mice were anesthetized with avertin, intubated using an i.v. catheter, and then provided with a total volume of 62.5μl containing 5 × 10^6^ plaque-forming units (PFUs) of adenoviral Cre through a micropipette inserted into their pharynx.

The mice utilized in this study were housed within a pathogen-free environment at Shanghai Tenth People's Hospital in Shanghai, China. Ethical guidelines for animal care and use, as set forth by the Institutional Animal Care and Use Committee of the hospital, were strictly adhered to during their treatment.

### Nude mouse xenograft model

Male BALB/c nude mice, aged four weeks, obtained from Shanghai Lingchang Biotechnology (Shanghai, China), were acclimatized for one week under specific pathogen-free conditions. Throughout the acclimatization period, mice were given unlimited access to standard chow and water. To assess the *in vivo* proliferation effect of STAT3 and ACC1, A549shControl, A549shSTAT3, and A549shACC1 cells were subcutaneously inoculated into the right flanks of these mice (n = 4 per group). Tumor dimensions were measured at regular intervals of 7-10 days using a digital caliper. Tumor volume was calculated as V = (a × b^2^)/2, where 'a' and 'b' represent the maximum and minimum diameters, respectively. After a duration of 30 days, the animals were euthanized, and the tumor masses were promptly recorded. The research protocols were approved by the Research Ethics Committee and complied with the guidelines established by the Animal Care and Use Committee of the hospital.

### Cell lines and culture

The NSCLC cell lines, H1299 and A549, derived from the American Type Culture Collection (ATCC) in the USA, were sustained in 1640 medium supplemented with 100 units/ml penicillin-streptomycin and 10% fetal bovine serum (FBS). These cells were incubated at 37 °C, in a humidified environment supplemented with 5% CO_2_.

### Lentivirus infection

The lentiviruses used in this study include the STAT3-silencing lentivirus (shSTAT3), the ACC1-silencing lentivirus (shACC1), and the negative control lentivirus (shControl), which were all provided by GENECHEM in Shanghai, China. Detailed sequence information for each lentivirus can be found in **Table S1**. To carry out the experiments, H1299 and A549 cells were initially seeded in 24-well plates and allowed to grow overnight. Subsequently, these cells were infected with lentiviral particles and polybrene (10 μg/mL, Beyotime Biotechnology, Shanghai, China). After the infection, the cells were selected by using puromycin (Beyotime Biotechnology, Shanghai, China) and were then harvested for further experiments. This selection process allowed us to ensure that only the desired cells were used for subsequent analyses.

### Western blot

After subjecting the cells to lysis using RIPA buffer (P0013B, Beyotime), SDS-PAGE was performed. The resulting lysates were then transferred onto PVDF membranes for subsequent immunoblotting analysis.

### RT-qPCR

To isolate RNA, the EZ-press Kit (B0004D, EZB) was employed, followed by reverse transcription utilizing the Prime Script RT kit (R333-01, Vazyme). Amplification was carried out using the SYBR Green Master Mix (11184ES08, YEASEN), using GAPDH as the internal control. To minimize redundancy, the language has been reorganized and revised.

### Immunohistochemistry

To investigate the specific antigens, paraffin-embedded tissues were probed using specific antibodies. In parallel, cells grown on coverslips were fixed, permeabilized, and subsequently incubated with the designated antibodies.

### Cell viability and Edu assay

Cells were plated in triplicate onto 96-well plates at a density of 2 × 10^3^ cells per well and allowed to adhere overnight in 1640 medium (RPMI 1640, Sigma-Aldrich). The cells were then cultured for various time points. Prior to detection, we added CCK-8 reagent (C0037, Beyotime Biotechnology Co., Ltd., Shanghai, China) and incubated it at 37 °C for 0.5 hours. The absorbance was then measured at 450nm using a microplate reader (Bio-Rad, USA). To label the dividing cells' nuclei, we performed Edu (C0071S, BeyoClick™ EdU, Beyotime) staining according to the manufacturer's instructions. Specifically, the cells were exposed to 10 μM Edu for 2 hours, fixed, incubated with DAPI, and finally observed under a laser scanning microscope.

### Transwell assay

In accordance with a previous study 20, we employed Transwell analysis to detect the migratory behavior of non-small cell lung cancer (NSCLC) cells. To facilitate the cell invasion assay, the upper compartment of the chamber was coated with a polycarbonate layer pre-coated with a matrix gel (40185ES08, Yeasen).

### Wound-healing assay

The migratory capacity of non-small cell lung cancer (NSCLC) cells was assessed using both wound-healing and Transwell assays. To conduct the wound-healing assay, 5 × 10^6^ H1299 and A549 cells were seeded in 12-well plates, and a scratch was created using a sterile pipette tip. Subsequently, the cells were cultured in serum-free 1640 medium, and the size of the wound was measured at both 0 and 24 hours.

### Colony formation assay

In the colony formation assay, 500 cells were seeded in each well of 12-well plates. After observable colony formation, the cells were washed using phosphate-buffered saline (PBS) and then stained with a 0.1% crystal violet solution for 5 minutes. Subsequently, the colonies were quantified.

### Chromatin immunoprecipitation assay

A549 cells underwent crosslinking with a 1% formaldehyde solution and subsequent lysis, utilizing buffers from the BeyoChIP™ Enzymatic Chromatin Immunoprecipitation (ChIP) Assay Kit with Protein A/G Magnetic Beads (P2083S, Beyotime). The resulting chromatin was then sonicated to generate fragments measuring approximately 200-300bp. Immunoprecipitation ensued, employing 5µg of a polyclonal anti-STAT3 mouse antibody (9139S, CST) or mouse IgG as a control. The antibody complexes were subsequently eluted and subjected to decrosslinking for DNA purification. To enable qPCR analysis, specific primers (Supplementary Table S1) were designed to amplify regions every 200bp within the 2000bp upstream region of the ACC1 promoter. RNA was extracted using the EZB-press RNA Purification Kit (B0004D, EZB). The amount of precipitated DNA was then quantified through RT-PCR and normalized against the input DNA.

### Dual-luciferase reporter assay

The promoter sequences of the human ACC1 gene were cloned into the GV238 vector. Subsequently, we transfected the constructed plasmid, consisting of a reporter plasmid (1 μg), a STAT3 overexpressing vector (1 μg), or a negative control (NC) vector (1 μg) with Renilla luciferase, into H1299 cells using Lipofectamine 8000. Following 28 hours of cell culture in a 12-well plate, we measured the levels of firefly and Renilla luciferase activity using the Dual-Luciferase Reporter Assay System (RG016, Beyotime), following the recommended protocol provided by the manufacturer. The luciferase activity was quantified using a microplate reader (Bio-Rad, USA). To assess the relative firefly luciferase activity, we normalized it to the Renilla luciferase activity, and subsequently calculated the fold changes in the reporters.

### Immunofluorescence

H1299 cells were cultured on a slide and subsequently fixed with 4% PFA. After permeabilization using 0.5% Triton X‐100/PBS, the cells underwent a 1-hour blocking step with 5% normal goat serum at room temperature. Following this, the cells were incubated with Nile red (1 µm) at 37°C for 30 minutes to label them. For visualization of the cell nuclei, 4,6-diamino-2-phenylindole (DAPI) was added and allowed to stain for 5 minutes. Finally, a confocal microscope (ZEISS, Germany) was used to capture the images.

### ATP production assay

In order to measure ATP production, we utilized the ATP Assay Kit (S002, Beyotime), adhering to the manufacturer's instructions.

### Gene Expression Data and Subsequent Processing Based on TCGA Database

The TCGA database, known as one of the most extensive repositories of cancer-related genetic information, encompasses a wide array of data types, including gene expression profiles, miRNA expression data, copy number variations, DNA methylation patterns, SNPs, and various other datasets. For our research, we obtained and processed mRNA expression data from 539 primary NSCLC specimens 21.

### Statistical analysis

The experimental results were analyzed using GraphPad Prism 9 (GraphPad Software Inc., San Diego, CA, USA). The results are presented as the mean ± standard deviation (SD). The statistical significance between two groups was assessed using Student's t-test. A p-value of less than 0.05 was considered as statistically significant.

## Results

### STAT3 promotes lung cancer cell proliferation and migration

**Figure 1:**
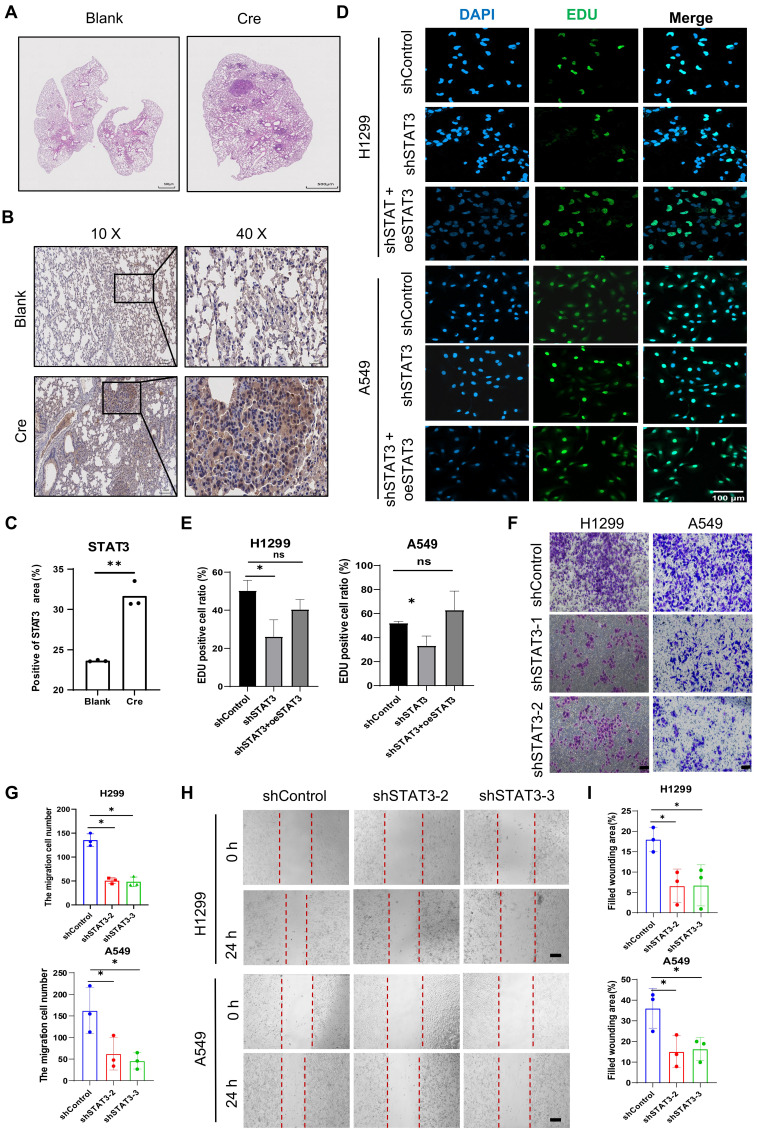
** STAT3 Modulation and Function in LUAD Model and NSCLC Cells. A.** Hematoxylin and eosin (H&E) staining was performed on lung tissue sections obtained from distinct groups of mice. This study utilized a lung adenocarcinoma (LUAD) model created by introducing conditional alleles of Kras^LSL-G12D^; p53flox/flox in mice (known as KP mice). The mice were stratified into two cohorts: one group received treatment with adenovirus expressing Cre recombinase (Cre), and the other group received control adenovirus (Blank). Scale bar, 500μm. **B-C.** Representative immunohistochemistry staining of the expression of STAT3 in lung sections from the mice as in **Figure 1A**. The quantification data are shown in (**C**). Scale bar, 100μm. **D-E.** Lung cancer cell line H1299 and A549 were used to establish stable STAT3 knockdown cell lines. Cell proliferation was examined by Edu. The Edu assay result showed that STAT3 overexpression rescued the proliferation ability insufficient in STAT3 knockdown cells. The quantification data are shown in (**E**). Scale bar, 100μm.** F-G.** Transwell assay were performed to evaluate the migration ability of H1299 and A549 cells after STAT3 knockdown. The quantification data are shown in (**G**). Scale bar, 100μm.** H-I**. Wound healing assay were performed to evaluate the migration ability of H1299 and A549 cells after STAT3 knockdown. Scale bar, 200μm. *P < 0.05, **P< 0.01, *** *P* < 0.001.

To investigate the role of STAT3 in lung cancer, we utilized a mouse model of lung adenocarcinoma with conditional alleles of Kras^LSL-G12D^ and p53^fl/fl^ **(Figure 1A)**. Our objective was to assess the expression of STAT3 in non-small cell lung cancer (NSCLC). To achieve this, we engineered mice with a conditional Kras mutation and a floxed p53 allele. The Cre-recombinase system was then used to activate the Kras mutation and delete the p53 gene, resulting in the spontaneous development of lung cancer in the mice 22. Immunohistochemical analysis revealed higher expression of STAT3 in the tumorigenic group induced by Cre compared to the control group **(Figure 1B-C)**. To further investigate the impact of STAT3 on NSCLC cells *in vitro*, we established NSCLC cells with stable knockdown of STAT3 using a shRNA lentiviral system **(Figure S1A)**. Subsequent Edu assays demonstrated that reducing STAT3 expression significantly inhibited the proliferation of NSCLC cells. Conversely, overexpressing STAT3 counteracted this inhibitory effect **(Figure 1D-E)**. Furthermore, migration assays using Transwell and wound healing techniques showed impaired migratory ability in H1299 and A549 cells lacking STAT3** (Figure 1F-I)**.

### STAT3 Regulates Lipid Metabolism in NSCLCs

**Figure 2:**
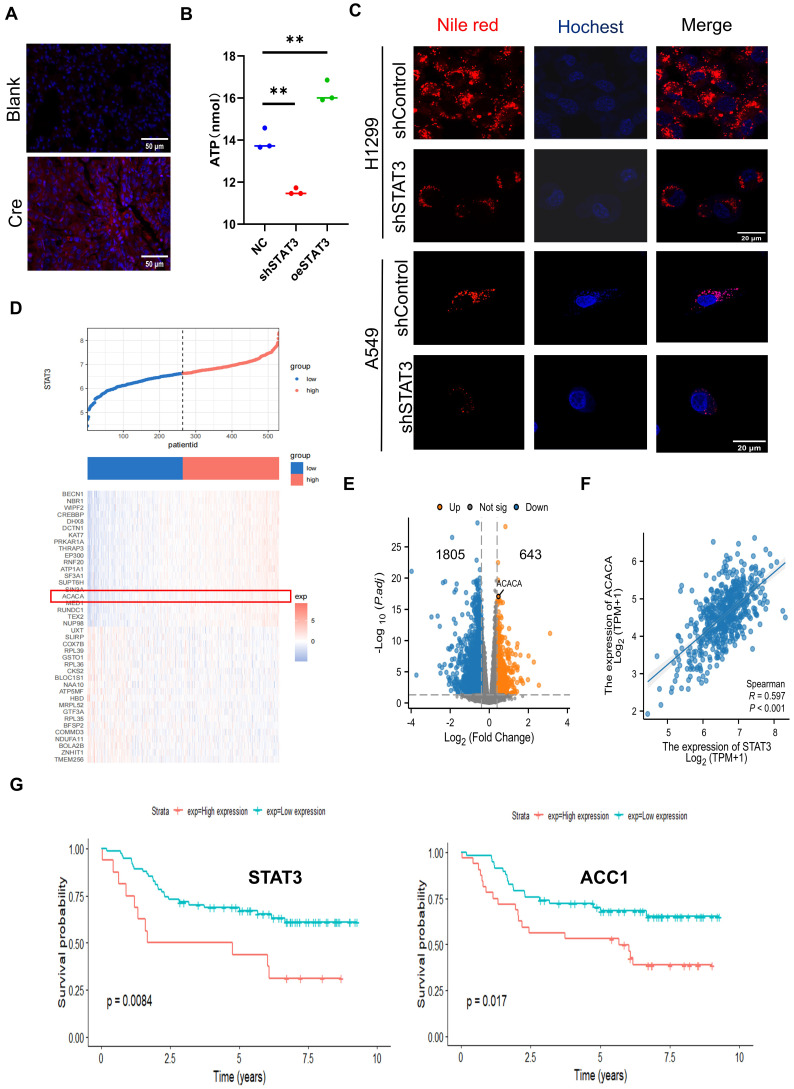
** Identification of Downstream Targets Regulated by STAT3 in Lipid Metabolism. A.** Representative immunohistochemistry staining of Nile red in section of lungs from mice as in **Figure 1A**. Lipids were stained with Nile red. Scale bar, 50 μm.** B.** Intracellular ATP of H1299 cells with knockdown or overexpression STAT3.** C.** Representative images of lipids in the control and STAT3 knockdown H1299 and A549 cells. Scale bar, 20μm. **D.** Heat map of STAT3 co-expressed genes. **E**. The differential expression of genes in two groups categorized as either high or low STAT3 expression based on the median expression are shown on the volcano plot. **F.** The correlation analysis among STAT3 and ACC1 in NSCLC based on the TCGA database. **G.** Using the GSE14814 dataset, Kaplan-Meier survival curve analysis of patients with high or low STAT3 or ACC1 expression. *P < 0.05, **P< 0.01, *** *P* < 0.001.

Metabolic reprogramming is an essential characteristic of tumorigenesis, and there is growing evidence indicating the involvement of lipid metabolism alterations in NSCLC 23. Through Nile Red staining, a significant accumulation of lipids was observed in the lungs of Cre-induced tumorigenic mice, suggesting a potential role of lipids in this context **(Figure 2A)**.

To explore the biological function of STAT3 in relation to this, we conducted a comparative analysis of ATP and lipid levels, revealing a decrease in both parameters upon STAT3 knockdown in NSCLC cells** (Figure 2B-C)**. These results indicate that STAT3 regulate lipid metabolism in NSCLC cells. To further explore the molecular pathways associated with STAT3 in lipid metabolism within NSCLC, we employed the TCGA plot package to identify genes co-expressed with STAT3 in NSCLC. The co-expression heatmap revealed genes that exhibited positive or negative correlations with STAT3 expression **(Figure 2D)**, among which we focused on ACACA (ACC1), a gene associated with lipid metabolism. The cellular lipid level is governed by the balance between synthesis and degradation. Notably, tumor cells often exhibit an elevated rate of FASyn 24. ACC1, a major isoform controlling FASyn, catalyzes the ATP-dependent carboxylation of acetyl-CoA to form malonyl-CoA. Additionally, we obtained the TPM expression matrix from The Cancer Genome Atlas (TCGA) for lung adenocarcinoma (TCGA-LUAD), comprising 539 lung cancer samples. These samples were stratified into STAT3 high-expression and low-expression groups based on the median STAT3 expression. The volcano plot showed that in the STAT3 high-expression group, ACC1 expression was significantly upregulated (|log2FoldChange|>4, P < 0.05)** (Figure 2E)**. Furthermore, analysis of the TCGA database revealed a positive correlation between STAT3 expression and ACC1 in lung cancer patients **(Figure 2F)**. Moreover, we performed Kaplan-Meier analysis to assess the prognostic value of STAT3 and ACC1 in lung cancer patients. The results indicated that lung cancer patients with higher STAT3 and ACC1 expression had significantly poorer overall survival (OS)** (Figure 2G)**. In summary, these findings suggest that ACC1 may serve as an effector of STAT3 in the regulation of lipid metabolism in lung cancer.

### ACC1 as a STAT3 Downstream Effector Modulating FASyn and Progression in Lung Cancer

**Figure 3:**
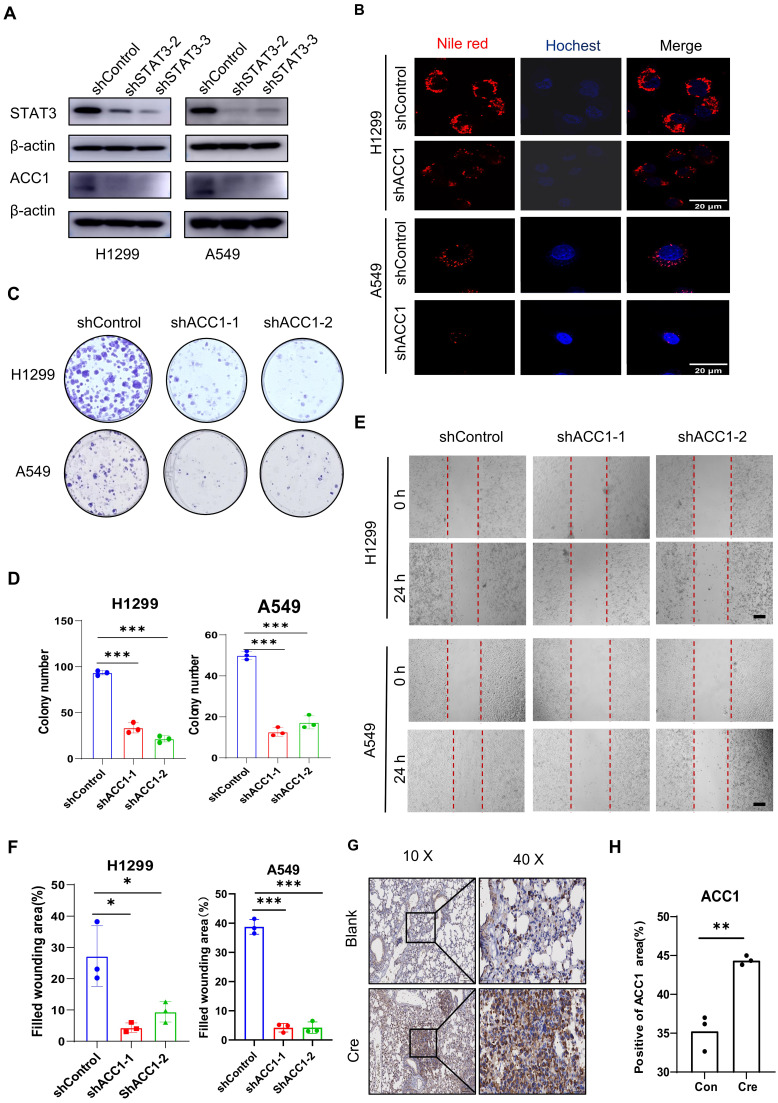
** ACC1 as a STAT3 Downstream Effector Modulating FASyn and Progression in Lung Cancer. A.** Expression of STAT3 and ACC1 was detected by Western blotting in H1299 and A549 cells transfected with shSTAT3 or control.** B.** Representative images of lipids contained in the negative control and ACC1 knockdown H1299 and A549 cells. Scale bar, 20μm. **C-D.** Colony formation assay was performed to evaluate the proliferation ability of H1299 and A549 cells after ACC1 knockdown. The quantification data are shown in (**D**). **E-F.** Wound healing assay was performed to evaluate the migration ability of H1299 and A549 cells after ACC1 knockdown. The quantification data are shown in (**F**). Scale bar, 200μm.** G-H.** Representative quantification immunohistochemistry staining of the expression of ACC1 in lung sections from the mice as in Fig 1A. The quantification data are shown in (**H**). Scale bar, 100μm. *P < 0.05, **P< 0.01, *** *P* < 0.001

Western blot analysis demonstrated a reduction in ACC1 expression after STAT3 knockdown in NSCLC cells** (Figure 3A).** Based on findings, we hypothesized that ACC1 may act as a downstream gene through which STAT3 regulates energy metabolism and lipid levels in lung cancer cells. Supporting this conjecture, Nile Red staining showed a decrease in lipid levels upon ACC1 knockdown in H1299 and A549 cells** (Figure 3B).** Moreover, functional assays involving colony formation and wound healing indicated that the absence of ACC1 compromised the proliferative and migratory capacities of H1299 and A549 cells **(Figure 3C-3F)**. Immunohistochemistry further confirmed an elevated ACC1 expression in the lung tissues of the Cre-induced group compared to the control group. In summary, these findings suggest that STAT3 may modulate ACC1 expression to regulate the proliferation and migration of lung cancer cells.

### Fatty acid synthesis is required in NSCLC cell proliferation and migration

**Figure 4:**
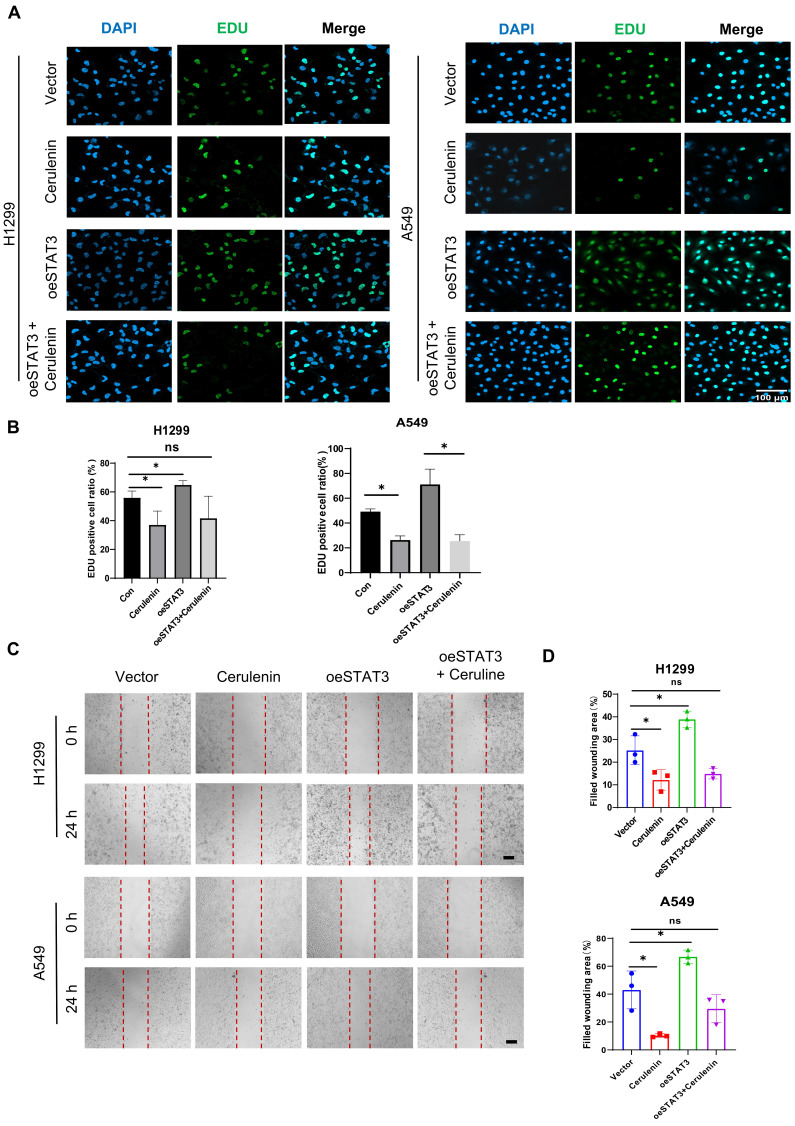
** FASyn inhibition suppresses proliferation and migration in NSCLC cells.** Vector and STAT3 overexpression plasmids were transfected into H1299 and A549 cells, followed by intervention with 10 μg/ml Cerulenin.** A-B.** Edu assay was performed to evaluate the proliferation ability of the cells in each group. The quantification data are shown in (**B**). Scale bar, 100μm. **C-D.** Wound healing assay was performed to evaluate the migratory capacity of cells in each group. The quantification data are shown in (**D**). Scale bar, 200μm. *P < 0.05, **P< 0.01, *** *P* < 0.001.

To further confirm the dependence of lung cancer cell proliferation and migration on lipid synthesis, we employed cerulenin (10μg/ml) as a non-competitive inhibitor of FAS. Cerulenin obstructs the condensation process of malonyl-CoA and acetyl-CoA, thus elongating fatty acids and inhibiting their synthesis 14. Our observations from the Edu assay and wound healing experiment provided compelling evidence for the significant suppression of NSCLC cell proliferation and migration following cerulenin treatment (**Figure 4A-4D**). Moreover, cerulenin effectively antagonized the pro-proliferative and pro-migratory effects conferred by STAT3 overexpression.

### STAT3 Promotes proliferation and migration by regulating ACC1 transcription in lung cancer

**Figure 5:**
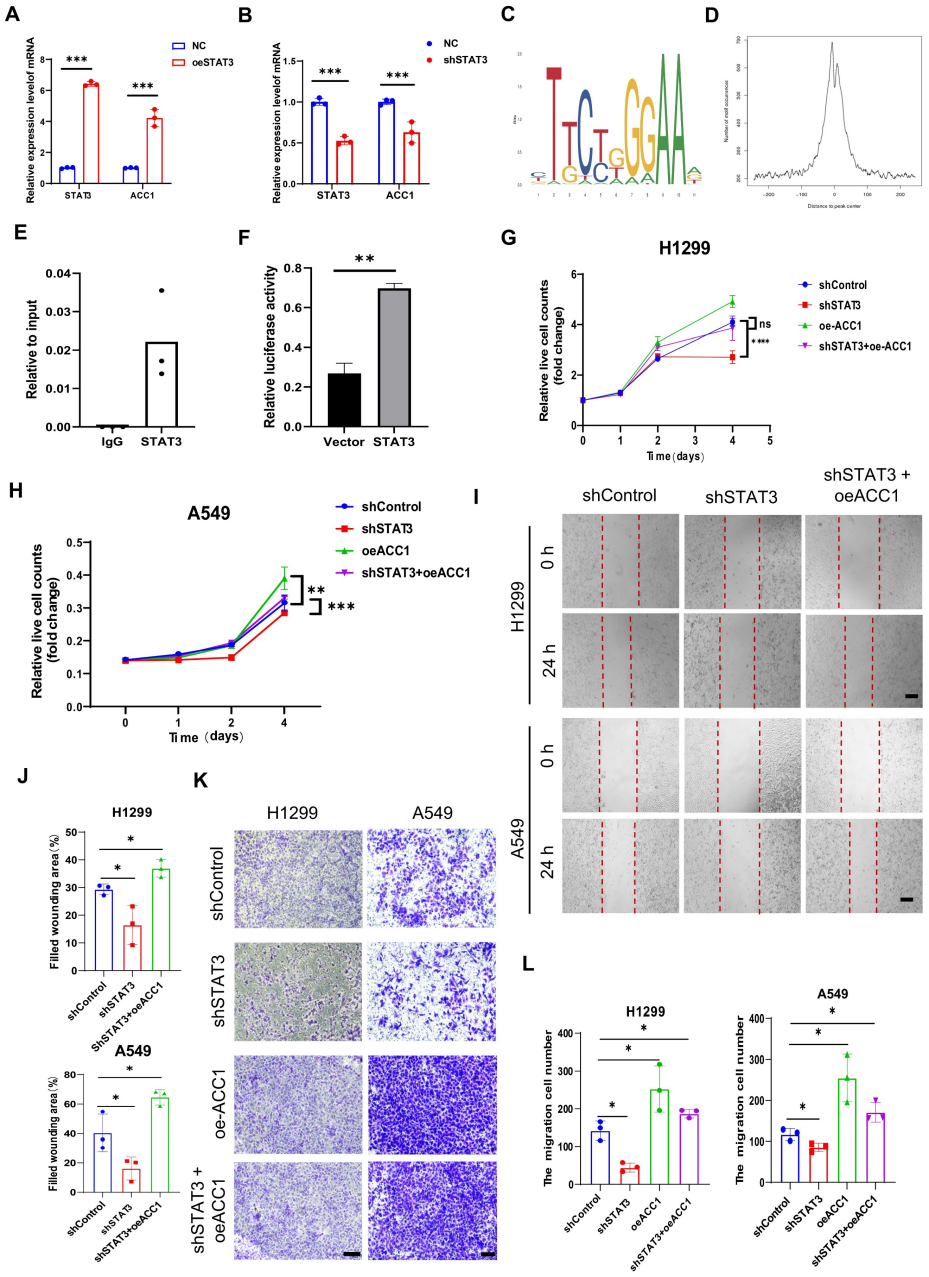
** STAT3 modulates proliferation and migration through Transcriptional Regulation of ACC1. A-B.** qRT-PCR showing the mRNA expression of STAT3 and ACC1 in STAT3-overexpression (**A**) or STAT3 knockdown (**B**) H1299 cells.** C-D.** Map of STAT3 binding site sequence. **E.** ChIP assay showing STAT3 binding to the ACC1 promoter in H1299 cells. The results were normalized to input DNA. Shown are mean ± SD (*n*=3). **F.** ACC1 promoter luciferase assay was conducted in H1299 cells 24 h after transfection with a control plasmid or a STAT3 overexpression plasmid. The luciferase activity was then normalized with control plasmid transfection. The relative firefly luciferase activity was normalized to renilla luciferase activity, and fold changes in the reporters were calculated.** G-H.** CCK8 assay was performed to evaluate the proliferation of H1299 and A549 cells in each group. **I-J.** Wound healing assay was performed to evaluate the migration ability of H1299 and A549 cells in each group. The quantification data are shown in (**J**). Scale bar, 200μm. **K-L.** Transwell assay was performed to evaluate the migration ability of H1299 and A549 cells in each group. he quantification data are shown in (**L**). Scale bar, 200μm. *P < 0.05, **P< 0.01, *** *P* < 0.001.

We conducted a comprehensive analysis to investigate the regulatory relationship between STAT3 and ACC1. Overexpression of STAT3 using a STAT3 overexpression plasmid led to an increase in ACC1 mRNA levels **(Figure 5A)**. In contrast, knockdown of STAT3 using shRNA in H1299 cells resulted in a decrease in ACC1 mRNA levels **(Figure 4B)**. Western blot analysis indicated that silencing STAT3 at the protein level also resulted in reduced ACC1 expression** (Figure 3A)**, suggesting that STAT3 may play a role in transcriptionally regulating ACC1 expression. To further explore this relationship, we performed a Chromatin immunoprecipitation (ChIP) assay, which revealed that STAT3 directly binds to the ACC1 promoter and regulates its expression in NSCLCs **(Figure 5C-E)**. Moreover, overexpression of STAT3 significantly increased the luciferase activity driven by the ACC1 promoter, demonstrating its transcriptional activation effect on ACC1 **(Figure 5F)**. To gain insights into the functional implications, we conducted ACC1 overexpression in NSCLC cells and observed that it counteracted the inhibitory effects of STAT3 knockdown on cell proliferation and migration** (Figure 5G-L)**. Collectively, these findings highlight the critical role of the STAT3-ACC1 axis in modulating the proliferative and migratory capacities of lung cancer cells.

### STAT3 and ACC1 upregulation in NCLCS predicts poor prognosis

**Figure 6:**
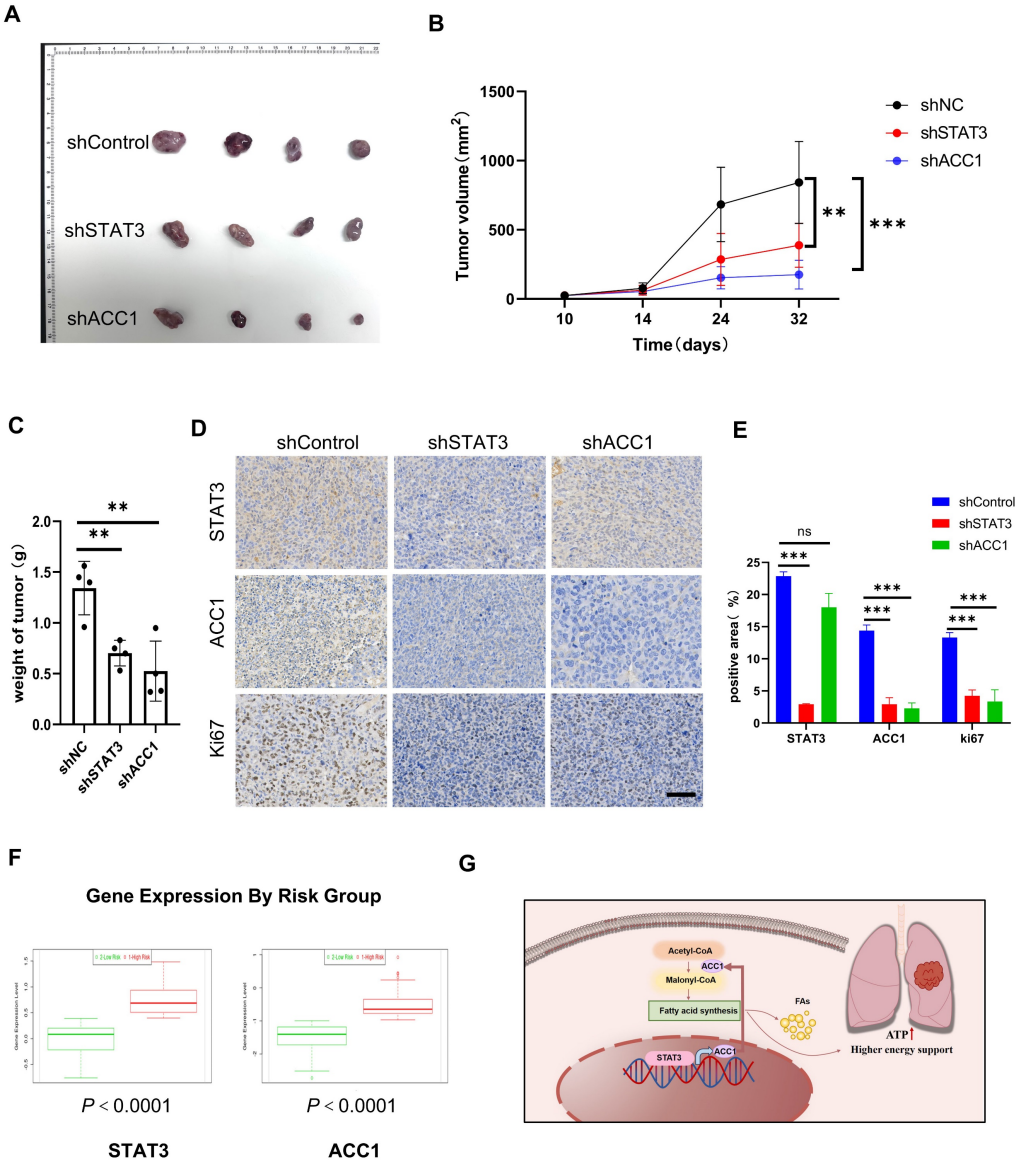
** Inhibiting STAT3 and ACC1 suppresses Lung Cancer Progression *in vivo*. A.** A549 cells from different experimental groups were subcutaneously injected into Balb/c Nude Mouse, and after one month the transplanted tumors were collected. **B.** Tumor volume was measured weekly in different groups.** C.** Tumor weight was measured in different groups(n=4). **D-E**. Histopathology of xenograft tumors stained with STAT3, ACC1 and Ki67. Scale bar = 20 μm. The quantification data are shown in (E). **F.** Using the GSE13213 lung cancer patient dataset, lung cancer patients were stratified into high-risk and low-risk groups based on their prognosis, and a comparative analysis of the relative mRNA expression of STAT3 or ACC1 was performed between the two groups.** G**. The mechanism diagram was generated to illustrate theSTAT3-ACC1-FAS axis in NSCLC. *P < 0.05, **P < 0.01, *** P < 0.001.

We conducted further investigations to examine the impact of STAT3 and ACC1 on the progression of lung cancer *in vivo*. A549 cells with stable knockdown of STAT3 and ACC1 were implanted subcutaneously into nude mice. Tumor volumes were measured on a weekly basis. The results clearly demonstrated that the silencing of both STAT3 and ACC1 led to a significant inhibition of tumor growth **(Figure 6A-6C)**. In addition, knocking down STAT3 significantly decreased STAT3, ACC1 and Ki-67 expression and knocking down ACC1 decreased ACC1 and Ki-67 as visualized by IHC staining. These results suggest that STAT3 could regulate the proliferation of NSCLCs via ACC1 **(Figure 6D-6E)**. To evaluate the prognostic significance of these identified markers in predicting patient survival, we utilized SurvExpress, an online tool that analyzes gene expression data from 502 TCGA cancer studies 25. This analysis examined the expression levels of both STAT3 and ACC1 in two low-risk groups and one high-risk group, indicating an upregulation of STAT3 and ACC1 expression in the high-risk group. This observation may suggest a potential association between the expression levels of STAT3 and ACC1 and patient prognosis** (Figure 6F)**. These results indicate that STAT3 and ACC1 may serve as novel prognostic markers for lung cancer.

## Discussion

In this study, we have uncovered a previously unidentified pathway involving STAT3-ACC1 axis, which significantly impacts lipid metabolism in lung cancer. Moreover, our investigation has revealed the integral role of this pathway in both the proliferation and migration of NSCLC cells. It is widely recognized that cancer cells undergo metabolic reprogramming to meet their augmented biosynthetic demands 26, 27. However, existing research has primarily concentrated on exploring glucose and glutamine metabolisms, thereby overlooking the crucial contribution of lipid metabolism 28, 29. Consequently, our findings provide novel evidence that underscores the critical involvement of fatty acid synthesis in the development and progression of tumors.

In addition to supporting the growth and migration of NSCLC cells, fatty acid synthesis (FAS) has been found to have significance in cancer invasion 30, drug resistance 31, and stem-like characteristics 32. Research has shown that mutant KRAS lung cancer relies on newly synthesized fatty acids to evade ferroptosis, presenting a targetable vulnerability 24. Based on a recent study, FAS inhibitor cerulenin could inhibit the mTOR signaling pathway, and suppressed cancer cell proliferation and migration 33. In our study, we investigated the effects of cerulenin on NSCLC cells and found that inhibiting FAS reduced lipid droplet accumulation. This study highlights the key enzyme ACC1 as a critical protein in FAS for NSCLC cell growth and migration. We also observed abnormally high expression of both STAT3 and ACC1 in the Kras^G12D/+^; p53^-/-^ mouse model of lung *in situ* carcinoma. Previous research has shown that suppressing ACC1 expression effectively restrains fatty acid synthesis and the proliferative growth of NSCLC cells 12. However, the upstream regulation of ACC1 has not been clearly elucidated.

Our study found that The STAT family proteins, particularly STAT3 and STAT5, are well-known for their vital contributions to various aspects of tumor progression, including cell proliferation 34, apoptosis resistance 35, promotion of carcinoma stemness, and development of drug resistance 36. In the context of cancer cell metabolism, STAT3 exerts regulatory control over numerous processes, including aerobic glycolysis 37, oxidative phosphorylation 38, generation of reactive oxygen species (ROS) 39, mitochondrial energy production, and modulation of mitochondrial membrane integrity 19. Previous research has highlighted the role of STAT3 in facilitating lipid transport via CD36, thus supplying ample energy for tumor cell proliferation and migration 40. Building upon these findings, our study provides further insights into the involvement of STAT3 in promoting lipid synthesis, thereby bolstering the evidence substantiating the modulation of lipid metabolism by STAT3 as a contributing factor to tumor cell growth.

According to our study results as well as TCGA database, STAT3 was upregulated in NSCLC and positively correlated with ACC1. We conducted cellular experiments to validate the regulatory effect of STAT3 on ACC1 at the transcriptional level. Moreover, we observed that overexpressing ACC1 partially reversed the inhibitory effect of downregulating STAT3 on proliferation, and migration in NSCLC. To further support our findings, we performed a xenograft experiment using A549 cells. The experiment revealed that low STAT3 and ACC1 expression effectively halted tumor growth. These findings highlight the potential of targeting the STAT3-ACC1 axis as a novel strategy for NSCLC treatment. However, our study primarily relied on specific lung cancer cell lines and animal models, which might limit the generalizability of our discoveries. Moreover, these models may not fully capture the complexity and heterogeneity of human lung cancer, nor the influences of the tumor microenvironment. Consequently, the direct applicability of these findings to human patients still needs to be further verified.

In recent times, there has been significant progress in the development of small molecule drugs that specifically target abnormalities in lipid metabolism, with a particular focus on inhibiting de novo lipid synthesis. Preclinical studies on small-molecule drugs targeting lipid metabolism, such as FASN inhibitors C75 and cerulenin 41, 42, ACLY inhibitors SB-204990 43, SREBP inhibitors fatostatin, PF-429242 44, 45, CD36-targeted drugs ABT-510 46, have been conducted. Nevertheless, their pharmacological properties and side effects limit their potential for clinical application. As such, it is crucial to develop tailored small-molecule drugs that effectively address abnormal lipid metabolism in tumor cells while minimizing undesirable side effects. This study, utilizing public databases, demonstrated that elevated expression of STAT3 and ACC1 in lung cancer patients is associated with higher risk and poorer prognosis. These research findings present an opportunity to identify potential metabolic targets for further development of experimental targeted small-molecule drugs.

In conclusion, this study emphasizes the significance of the STAT3-ACC1-FAS pathway in non-small cell lung cancer (NSCLC). Notably, ACC1 activation through STAT3 transcription serves as a critical mechanism driving tumor progression and lipid synthesis in NSCLC, as depicted in **Figure 6G**. Consequently, targeting the STAT3-ACC1 axis holds great potential as a novel and promising diagnostic and therapeutic strategy for NSCLC patients.

## Supplementary Material

Supplementary figure and table.

## Abbreviations

STAT: signal transducer and activator of transcription
ACC1: acetyl‐CoA carboxylase1
FAS: fatty acid synthase
FAs: fatty acids
NSCLC: non-small cell lung cancer
IF: immunofluorescence
ChIP: chromatin immunoprecipitation
IHC: immunohistochemistry
HE: hematoxylin‐eosin
ATP: adenosine triphosphate
