# Supplementary material for: Unveiling the STAT3-ACC1 axis: a key driver of lipid metabolism and tumor progression in non-small cell lung cancer
Source: J Cancer. 2024 Mar 4;15(8):2340–53. doi: 10.7150/jca.93890 (PMC10937262; doi:10.7150/jca.93890)
Supplement: Supplementary file 1 — Supplementary figure and table. [file jcav15p2340s1.pdf]

Supplementary figures and tables

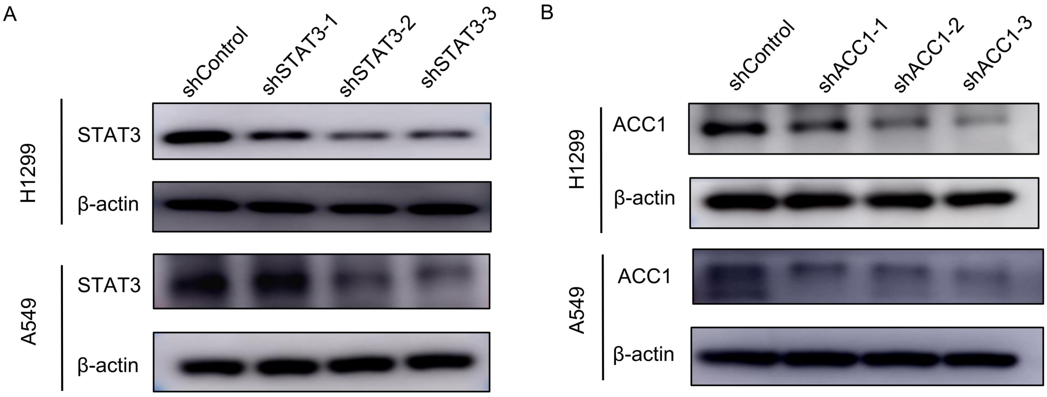

Fig.S1 A. The protein level of STAT3 was detected in STAT3 knockdown H1299 and A549 cells by western blot. **B.** The protein level of ACC1 was detected in ACC1 knockdown H1299 and A549 cells by western blot.

Supplementary Table S1. The sequence of indicated primers, shRNA used in this study.

**Name**		**Sequence (5'-3')**
**qPCR primer**		
STAT3	Forward	CTTTGAGACCGAGGTGTATCACC
	Reverse	GGTCAGCATGTTGTACCACAGG
ACC1	Forward	TTCACTCCACCTTGTCAGCGGA
	Reverse	GTCAGAGAAGCAGCCCATCAC
GAPDH	Forward	AGAAGGCTGGGGCTCATTTG
	Reverse	AGGGGCCATCCACAGTCTTC
**shRNA**		
shSTAT3-1	Target Sequence	CGGAGAAGCATCGTGAGTGAG

shSTAT3-2	Target Sequence	AAATGAAAGTGGTAGAGAATC
shSTAT3-3	Target Sequence	TGGAAACAACCAGTCAGTGAC
shACC1-1	Target Sequence	GGTCACACCTGAAGACCTTAA
shACC1-2	Target Sequence	GCAGATCTTAGCGGACCAATA
shACC1-3	Target Sequence	GCTTTCAGATGAACAGAATTG
**ChIP assay (Distance from ACC1, bp)**		
ACC1-prom-1755-1985	Forward	CCATCTCCACCCCTGTTG
	Reverse	CCTCAATTTGGGCCTCTG
ACC1-prom-1575-1773	Forward	GAGGACTGGGAGGATGGG
	Reverse	GCAACAGGGGTGGAGATG
ACC1-prom-1398-1593	Forward	GCCTCCGGGCTTTGCGTG
	Reverse	CCCCATCCTCCCAGTCC
ACC1-prom-1199-1416	Forward	CAGCCTGCCGCGTCAAG
	Reverse	GCACGCAAAGCCCGGAG
ACC1-prom-1063-1295	Forward	GCTGAGCGGGAAATGGC
	Reverse	CCTCTAGCGGGACTAGGGAG
ACC1-prom-862-1081	Forward	CGCAGGCTCCCGAGTTGT
	Reverse	TCGCCATTTCCCGCTCA
ACC1-prom-723-890	Forward	GAGACCACATTCTGACACCA
	Reverse	ACGTTGGCTCCACAACTC
ACC1-prom-518-748	Forward	ACCCCACCTCTGCTGAA
	Reverse	GGAAGTTGGTGTCAGAATGTG
ACC1-prom-322-534	Forward	GGAGGCAGAAGTTGCAGTG
	Reverse	TTCAGCAGAGGTGGGGTT
ACC1-prom-157-392	Forward	AGTCCCAGGTGGGCAGAT

	Reverse	TGAGATGGCGTTTCTTTCG
ACC1-prom-57-182	Forward	CAGTAAAGGATGCACAGTCAATA
	Reverse	CTCAGGTTATCTGCCCACC
